# Breakdown of accommodation in nerve: a possible role for persistent sodium current

**DOI:** 10.1186/1742-4682-2-16

**Published:** 2005-04-12

**Authors:** Kristian Hennings, Lars Arendt-Nielsen, Ole K Andersen

**Affiliations:** 1 Center for Sensory-Motor Interaction (SMI), Aalborg University. Frederik Bajers Vej D3-203, 9220 Aalborg Ø, Denmark

## Abstract

**Background:**

Accommodation and breakdown of accommodation are important elements of information processing in nerve fibers, as they determine how nerve fibers react to natural slowly changing stimuli or electrical stimulation. The aim of the present study was to elucidate the biophysical mechanism of breakdown of accommodation, which at present is unknown.

**Results:**

A model of a space-clamped motor nerve fiber was developed. It was found that this new model could reproduce breakdown of accommodation when it included a low-threshold, rapidly activating, persistent sodium current. However, the phenomenon was not reproduced when the persistent sodium current did not have fast activation kinetics or a low activation threshold.

**Conclusion:**

The present modeling study suggests that persistent, low-threshold, rapidly activating sodium currents have a key role in breakdown of accommodation, and that breakdown of accommodation can be used as a tool for studying persistent sodium current under normal and pathological conditions.

## Background

Accommodation is important for information processing in nerve fibers, as it determines whether, and how frequently, slowly-changing natural and artificial stimuli are translated into action potentials. Hill's theory of accommodation in nerve has been one of the most influential theories in this area [1]. A prediction of this theory is that a linearly rising current requires a certain critical slope in order to excite nerve fibers. Although this critical slope has been demonstrated in experimental preparations [2,3], it has not been found under normal physiological conditions [4,5]. Instead, nerve fibers have been shown to exhibit breakdown of accommodation; that is, a long-duration slowly rising current excites nerve fibers at a nearly constant intensity no matter how slowly this intensity is approached [4,5]. A critical slope has only been found for depolarized nerve fibers, and Hill's theory of accommodation has been shown only to be applicable to such fibers [6]. Accommodation and breakdown of accommodation were the foci of several studies before the invention of the voltage-clamp, since prior to this innovation it was one of the few methods by which membrane kinetics could be studied. Since the invention of the voltage-clamp and later the patch-clamp some fifty years ago, the concept of breakdown of accommodation has been virtually absent from the scientific literature [7]. However, the biophysical mechanism responsible for breakdown of accommodation is still unknown; and as will be shown in this paper, a model that only contains transient sodium channels (i.e. currents that activate and deactivate rapidly in response to membrane depolarization) is unable to reproduce the phenomenon. Persistent (no inactivation) and late (slow inactivation) sodium channels have been identified in large dorsal ganglion neurons [8] and it has been found that these channels are needed for modeling latent addition in motor and sensory nerve fibers (i.e. threshold changes to short sub-threshold stimuli [9]). This suggests that persistent or late sodium channels are present in both motor and sensory myelinated nerve fibers and have fast activation kinetics that can initiate action potentials. The present study was undertaken to study the hypothesis that persistent sodium channels create a "threshold region" of membrane depolarization that cannot be exceeded without the generation of an action potential. Thus, it is suggested that persistent sodium channels are the cause of breakdown of accommodation.

The results in the present paper were based on a model of a space-clamped nerve fiber. This model included a persistent sodium channel based on the work of Bostock and Rothwell (1997) [9]. This channel was defined from the transient sodium channel with the following modifications: a) inactivation was removed (a persistent channel); b) the time-constant was slowed by a factor of two (time-constant); and c) the kinetics was displaced so that the channel was activated at a membrane potential 20 mV more negative than is required to activate the transient channel (voltage shift) [9].

## Results

### Model validation

**Figure 1:**
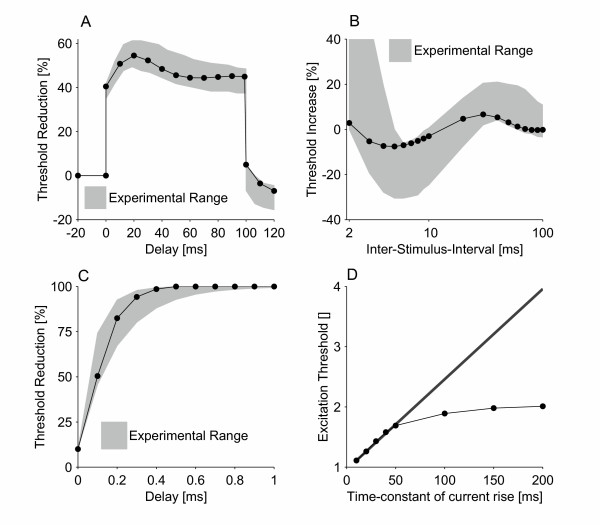
Comparison of the new model with experimental data for: A) threshold electrotonus [40], B) recovery cycle [41], C) latent addition [10], and D) accommodation curve [5]. In threshold electrotonus, a sub-threshold conditioning pulse of 100 ms duration is used to alter the threshold of a test stimulus delayed with respect to the onset of the conditioning pulse. In the recovery cycle, the nerve fiber is excited by a supra-threshold stimulus and the threshold of a test stimulus is determined at inter-stimulus intervals (T_ISI_) of 2 ms to 100 ms. In latent addition, a short duration sub-threshold conditioning stimulus is used to alter the threshold of a test stimulus; the onset of the test stimulus is delayed with regard to the onset of the conditioning stimulus. In the accommodation curve, the threshold of stimuli of the form I_S_(1-e-^tτ^) was determined, where τ was the time-constant of the current rise. In A, the bold line is the initial critical slope, which was estimated from the first four points in the accommodation curve where it is approximately a straight line. Experimental range: a) minimum and maximum of the experimental range, b) and c) mean ± standard deviation.

The structure of the model and the choice of parameters allowed it to reproduce four sets of independent experimental data: threshold electrotonus, recovery cycle, latent addition and breakdown of accommodation (see Figure 1). The model was found to have a strength-duration time constant of 133.2 μs, which is similar to the experimental recorded value for the median nerve (139 ± 59 μs [10]). Furthermore, the model simulated breakdown of accommodation (see Figure 1D). The initial critical slope of the model was found to be 17.3 rheobase/s, which is lower than the experimentally recorded value (21.2 ± 3.72 rheobase/s) for ulnar nerves. The breakdown of accommodation seen in the model was likewise greater than observed in the ulnar nerve, and was closer to that observed in sensory nerve fibers [5]. The accommodation curve flattened out and remained near 2 rheobases when the time-constant of the current rise was greater than ~150 ms.

### Breakdown of accommodation

**Figure 2:**
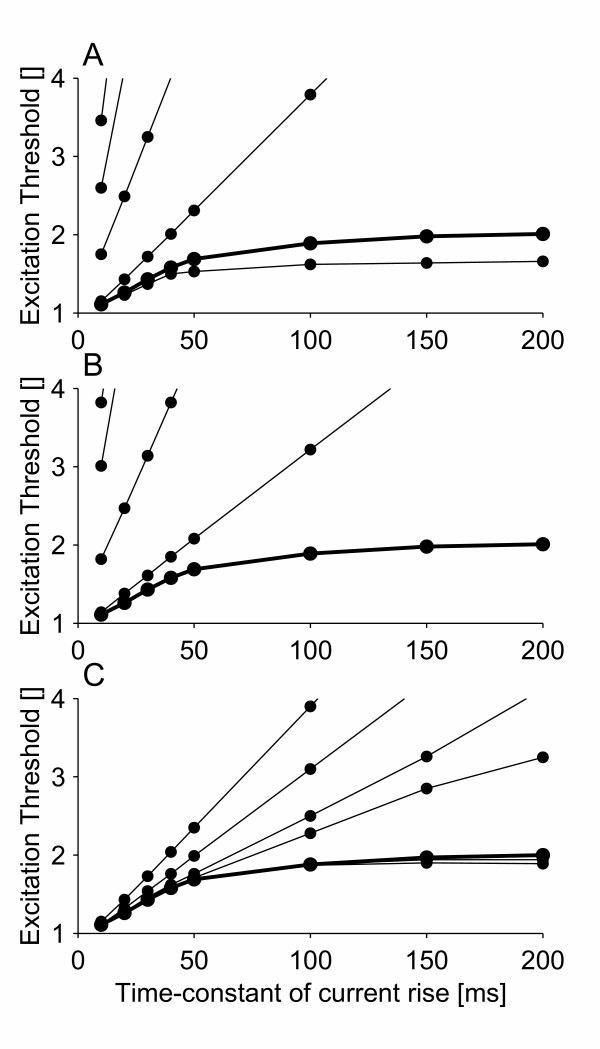
Relationship between the properties of persistent sodium channels and breakdown of accommodation: A) Number of persistent sodium channels (number of persistent sodium channels: 1.0%, 1.5%, 2.0%, **2.5%**, and 3.0%). B) Voltage shift of the kinetics of the persistent sodium channels relative to the transient sodium channels (voltage shift: -0 mV, -5 mV, -10 mV, -15 mV, and **-20 mV**). C) Time constant of persistent sodium channel activation (time-constant slowed by a factor of: 10, 6.66, 4.5, 3.0, **2.0**, and 1.0). A thick line and bold number indicates the default model.

**Figure 3:**
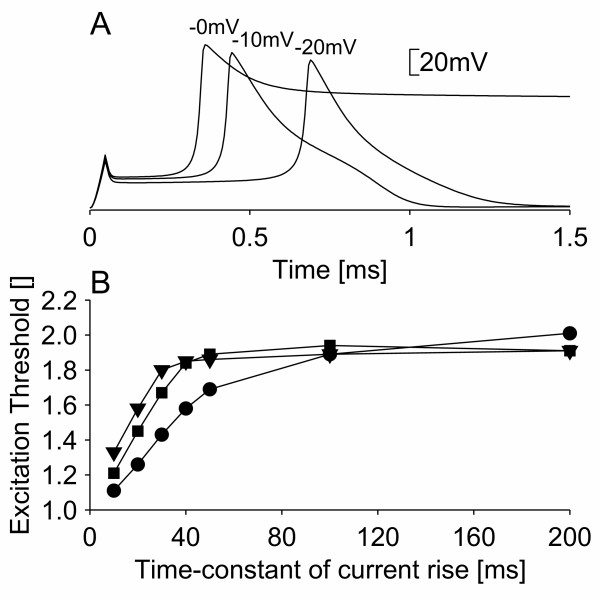
Relationship between voltage shift of persistent sodium channels relative to transient sodium channels and the shape of the action potential. For voltage shifts of -10 mV and -0 mV, the number of persistent sodium channels is set to a value (3.75% and 15%, respectively) that would produce approximately the same degree of breakdown of accommodation as the default model (voltage shift of -20 mV). A) The shape of the action potentials. B) The accommodation curves for the three voltage shifts of -20 mV●, -10 mV■, and -0 mV▲.

In order to study the relationship between the properties of persistent sodium channels and breakdown of accommodation, one parameter at a time (number of channels, voltage shift, time constant) was changed and its influence on breakdown of accommodation was assessed (see Figure 2). When the voltage shift was decreased from -20 mV to -10 mV or -0 mV, it was still possible to create breakdown of accommodation by increasing the number of persistent sodium channels to 3.75% (-10 mV) or 15% (-0 mV) of the total number of sodium channels (see Figure 3A). However, for a voltage shift of -0 mV, the membrane potential did not return to the resting potential after the generation of an action potential (see Figure 3A).

### Threshold responses to linearly rising stimuli

**Figure 4:**
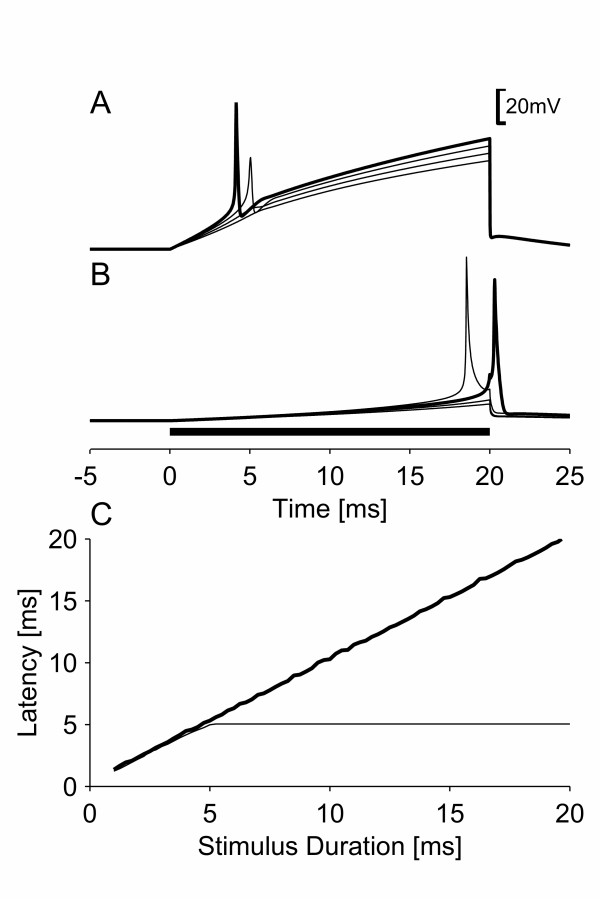
Responses of the new model without (A) and with (B) persistent sodium channels to a linearly rising current; (C) the latencies of the threshold responses for the models without (bold line) and with (thin line) persistent sodium channels. In subfigures (A) and (B) the responses for each model are shown for increasing stimulus intensities: A) 0.950, 0.975, 1.00, and 1.025 excitation threshold, and B) 0.925, 0.950, 0.975, and 1.00 excitation threshold. Threshold responses are drawn with bold lines.

The threshold responses to linearly rising stimuli were significantly modified by the presence of persistent sodium channels (see Figure 4). (A threshold response is a response to a stimulus with intensity equal to the excitation threshold of the nerve fiber). Without persistent sodium channels, the threshold response to a linearly rising current of 20 ms duration did not occur at the end of the stimulus but had a latency of 5.04 ms (see Figure 4A). With 2.5% persistent sodium channels, the threshold response occurred at the end of the stimulus (see Figure 4B). This difference was found for all linearly rising currents tested that had stimulus durations in the range 1 ms to 200 ms; when the model had 2.5 % persistent sodium channels the threshold responses were always observed at the end of the stimulus, whereas without persistent sodium channels the longest latency of the threshold response was 5.04 ms (see Figure 4C). For both models, with and without persistent sodium channels, a non-linear response always occurred when the membrane was depolarized to a certain threshold value. This non-linear response initially occurred at the end of the stimulus, and with persistent sodium channels it resulted in an action potential. Without persistent sodium channels, it only resulted in an action potential when the stimulus intensity was sufficient for the response to occur with a latency of 5.04 ms or less.

### Threshold electrotonus

**Figure 5:**
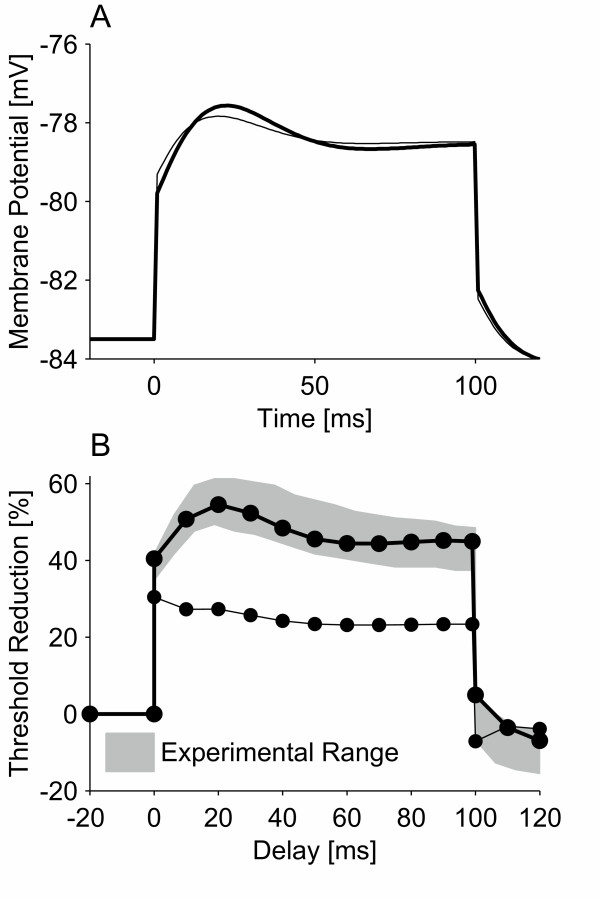
Electrotonus (A) and threshold electrotonus (B) of the new model with (thick line) and without (thin line) persistent sodium channels. The intensities of the conditioning currents were 40% and 30.5% of the threshold of the test stimulus alone for the model with and without persistent sodium channels, respectively.

A proportional relationship between the threshold change of a test stimulus and the underlying electrotonic changes in the membrane potential is a fundamental requirement for threshold electrotonus. Such a relationship was found for the model with 2.5% persistent sodium channels (see Figure 5). However, when the persistent sodium channels were removed from the model, the relationship between threshold and membrane potential broke down. This was tested with the conditioning current at an intensity of 40% for the model with persistent sodium currents, and 30.5% for the model without persistent sodium current. These two conditioning current intensities produced similar membrane depolarizations in the two models, which enabled the effect of the persistent sodium channels to be assessed. The relationship between threshold and membrane potential has also been found to break down when nerve fibers are depolarized because of a long-duration conditioning current or ischaemia [6]. Consequently, had the membrane depolarizations not been matched in the two models, the loss of the relationship between threshold and membrane potential may have been attributable to the greater membrane depolarization in the model without persistent sodium channels.

## Discussion

We have used a model of a space-clamped motor nerve fiber to provide evidence for a link between persistent sodium currents and breakdown of accommodation. The model demonstrated that these channels might be the cause of breakdown of accommodation, as their inclusion enabled the model to reproduce the phenomenon (see Figure 1D). It also demonstrated that such channels are likely to be low-threshold and rapidly activating (see Figure 2). The low-threshold property is further supported by the fact that although breakdown of accommodation can be reproduced by high-threshold persistent sodium channels, in this case it results in an action potential that does not return to the resting potential (see Figure 3).

### Experimental evidence for the role of persistent sodium current in breakdown of accommodation

Persistent, late sodium currents have been observed in large dorsal root ganglion cells. These current were found to have a low threshold and fast activation kinetics and were therefore expected to modulate membrane excitability by amplifying and prolonging depolarization from a generator potential or an external electrode [8,11]. Indirect evidence has been obtained for the presence of such channels in both large diameter sensory nerve fibers and motor nerve fibers [9], and they can produce regenerative currents that facilitate action potential generation. Persistent sodium channels have been shown to amplify otherwise sub-threshold depolarization, thereby initiating action potentials [12]. Furthermore, acidification and alkalization within the physiological range have been found respectively to decrease and increase persistent and late sodium currents [13]. This pH-dependence of the late sodium current correlates well with experimental observations of breakdown of accommodation. Hence, breakdown of accommodation has been found to decrease during ischaemia and increase during hyperventilation [5]. Furthermore, when nerve fibers are depolarized with a polarizing current, there is a decrease in the threshold to triangular stimuli [14]. This suggests that it is not membrane depolarization *per se *that causes loss of breakdown of accommodation and the presence of a critical slope for slowly rising stimuli. In the present study, the loss of breakdown of accommodation is explained by loss of the persistent sodium current, such as would be caused by ischaemic depolarization due to acidification. Consequently, the present study predicts that the critical slope found by [2] was caused by ischaemic acidification and not membrane depolarization.

### The effect of persistent sodium channels on threshold responses

In the present study, when the models with and without persistent sodium currents were stimulated by linearly rising currents, non-linear responses always resulted in action potentials when the model exhibited breakdown of accommodation. However, without breakdown of accommodation, non-linear responses only resulted in action potentials when they occurred within a "critical latency" from the onset of the stimulus. Without a "critical latency", which is the case with breakdown of accommodation, the threshold response occurred at the cessation of a long linearly rising stimulus. Consequently, the threshold for such stimuli is nearly constant regardless of their duration. However, when there is a "critical latency", the membrane potential needs to reach the voltage threshold within this "critical latency" for the nerve fiber to fire an action potential. The critical slope will then be proportional to the voltage threshold for which a non-linear response occurs divided by the "critical latency"; i.e. a decrease in "critical latency" results in an increased critical slope.

### Model limitations

The present model simplifies existing knowledge of neuronal morphology and the distribution of ion channels. With regard to ion channels, only two potassium channels and two sodium channels were included in the model, but at least five distinct potassium channels [15] and three persistent and late sodium channels [11] have been identified, besides the classical transient sodium channel [16]. Unfortunately, current knowledge of the potassium and sodium channels in motor nerve fibers does not provide enough detail to allow modeling of them all. For example, the channel densities and kinetics are not known for all five potassium channels [15], and the kinetic data we have for the slow and fast potassium channels are likely to represent amalgamations of several channel species into single stereotypes [16]. Consequently, the present model is based on an amalgamation of distinct channels into stereotypes and the detailed geometrical structures of motor nerve fibers into a gross equivalent electrical circuit. The parameters of the model were based on experimental current- and voltage-clamp recordings whenever possible and the results obtained were found to be in line with experimental work. Consequently, these simplifications appear justified and therefore provide a basis for studying the biophysical properties of breakdown of accommodation. This assumption is supported by previous work where models have provided insights into biophysical mechanisms [9,17,18]. However, the internodal leak resistance (R_IL_) in particular was not based on experimental data, but was instead set by trial and error to a value that would enable the model to reproduce known experimental data. This approach was used since few experimental data on the internodal leak resistance are available. There are only modeling data on the periaxonal resistivity [19], but further modeling data suggest that the longitudinal conductance of the myelin sheaths has to be taken into account in determining the internodal leak resistance [20]. Consequently, an internodal leak resistance based solely on the width of the periaxonal space is likely to be an underestimate. The unknown resistivity of the periaxonal space presents further difficulties in obtaining a value for internodal leak resistance on the basis of experimental data alone. For these reasons we believe that the present approach was justified.

### Alternative explanations of breakdown of accommodation

An alternative explanation for breakdown of accommodation could be the gating mode of the transient sodium channel [21]. The present paper follows the convention of assuming that activation and inactivation are two independent processes (i.e. the formalism of [22]). Today, it is known that activation and inactivation are inter-dependent, and that most transient sodium channels will go through an open state before entering an inactivated state [21]. This difference between the Hodgkin and Huxley formalism and recent knowledge of transient sodium channel function may have a synergistic role in breakdown of accommodation. Hence, a transient sodium channel with little inactivation before channel opening would not permit a critical slope and loss of breakdown of accommodation. However, this explanation remains unproven and would not change the conclusion of the present study, that persistent and late sodium channels can cause breakdown of accommodation. The interdependence of transient channel activation and inactivation may change the densities of persistent sodium channels needed for creating breakdown of accommodation, and thus there may be synergism between transient and persistent sodium channels.

A second explanation may be m-h overlap in the activation/inactivation kinetics of the transient sodium channel. For transient sodium channels, there is a region of membrane depolarization in which a persistent sodium current is generated l [23]. This is caused by channel activation while the membrane is still not sufficiently depolarized for all the channels to be inactivated, a phenomenon that has been termed m-h overlap. A theoretical study has demonstrated that the original squid axon model of Hodgkin and Huxley has breakdown of accommodation as a result of m-h overlap [23]. In this paper and other studies [9,24], persistent sodium channels are modeled as discrete channels. However, this does not imply that they are physically different from transient sodium channels. Three discrete persistent and late sodium currents have been identified on the basis of inactivation kinetics [11] in addition to the classical transient sodium current [16], but only one sodium channel Na_v _(1.6) has been found in the nodes of Ranvier in large peripheral nerve fibers [25]. This may suggest that persistent and late sodium currents are not generated specifically, but instead by transient sodium channels that operate in a gating mode with no or slowed channel inactivation. The modeling of persistent sodium current as created by persistent sodium channels does not provide evidence for the existence of such channels, only evidence that persistent sodium current can lead to breakdown of accommodation. Consequently, such persistent sodium current may be created by m-h overlap. However, in studies on persistent sodium currents, it has been argued that m-h overlap is not consistent with the observed kinetics [8,11]. Evidently, in mammalian nerve fibers, the persistent sodium current is most likely not generated by m-h overlap; but the study of [23] suggests that m-h overlap may be important for the persistent sodium current and breakdown of accommodation in squid axons.

## Conclusion

The present modeling study has demonstrated that persistent sodium currents can create a "threshold region" for membrane depolarization that cannot be exceeded without the generation of an action potential. Thus, a persistent sodium current may be the underlying biophysical mechanism for the breakdown of accommodation to slowly rising currents, which are observed under normal physiological conditions in mammalian nerve fibers [4,5]. This suggests that accommodation curves can be used as a tool for studying persistent sodium currents under normal and pathological conditions.

## Methods

### Electrical model of a motor nerve fiber

**Figure 6:**
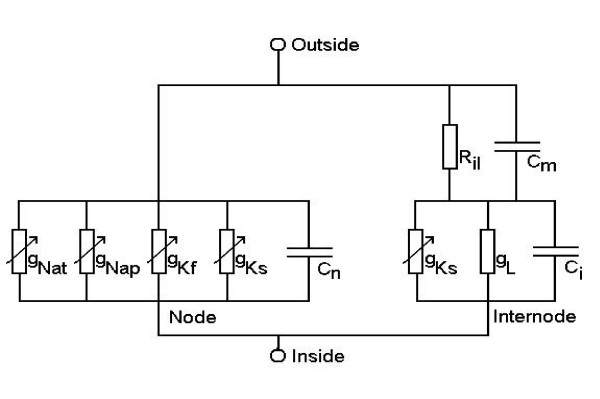
Equivalent circuit for a space-clamped motor neuron. The model consisted of a node and an internode. Both the node and the internode contained non-linear current sources, which were calculated from equilibrium potentials and conductances. Channel types and maximum ionic conductances: node, transient sodium (Na_t_, 276nS), persistent sodium (Na_p_, 7.1nS), fast potassium (K_f_, 4.1nS), and slow potassium (K_s_, 17.4nS); internode, slow potassium (K_s_, 87.1nS) and leak conductance (L, 1.7nS). The linear parameters of the model were: C_n_, nodal capacity (0.22pF), C_i_, internodal capacity (379pF), C_m_, capacity of the myelin sheath (0.17pF), and R_il_, internodal leak resistance (41 MΩ).

The structure of the model of the space-clamped motor nerve fiber was based on previous models used for studying the accommodative properties of such fibers [9,26,27]. The present model represents a motor nerve fiber by the electrical equivalent circuit shown in Figure 6. The geometry of the node and internode was based on studies on the morphology of cat ventral spinal roots. The geometrical parameters were taken from cats of 1–11 years of age for a motor nerve fiber with a diameter of 14 μm (see Table 1). The nodal, internodal and myelin capacitances in the electrical equivalent circuit were calculated on the basis of these geometrical parameters and experimentally estimated capacitances per square micrometer (see Table 2 and Figure 6). The internodal leak resistance (R_il_) and nodal resting potential were set by trial and error rather than calculated from geometrical and electrical parameters (see section entitled 'Validation', below).

**Table 1:** Geometrical parameters

Inter-nodal length (L)	1.37 mm	[45]
Inter-nodal diameter (d_i_)	8.8 μm	[46]
Nodal diameter (d_n_)	3.5 μm	[47]
Nodal length (*l*)	1 μm	[48]
Number of myelin lamella (N)	141	[46]

**Table 2:** Electrical parameters

Nodal capacitance (c_n_)	2 μF/cm^2^	[39]
Internodal capacitance (c_i_)	1 μF/cm^2^	[49]
Myelin capacitance (c_m_)	0.1 μF/cm^2^	[50]

### Ionic currents

Five major ionic currents have been identified in myelinated nerve fibers as necessary for modeling a wide variety of experimental data: the transient sodium current (Na_t_) for modeling the action potential [22], and the persistent sodium current (Na_p_) for modeling latent addition [9] and the recovery cycle [24]. Fast (K_f_) and slow (K_s_) potassium currents have been shown to explain accommodation to depolarizing conditioning currents [28]. Accommodation to hyperpolarizing currents can be explained by a hyperpolarization-activated cation conductance (I_H_), which is also thought to limit hyperpolarization in nerve fibers after they have conducted a train of impulses [28,29].

Transient and persistent sodium channels were included in the node, but following the work of [9] they were omitted from the internode for simplicity. The hyperpolarization-activated cation conductance was omitted from the model as it does not influence the response of nerve fibers to depolarizing stimuli [14]. Based on the work of [28,30,31]., the slow potassium current was included in the node as well as the internode. There is evidence for the localization of fast potassium channels in the paranode [32-35] As the paranode was not included in the present model, it was impossible to include fast potassium channels at this location. Instead, the approach used by [36] was applied and the fast potassium channels were included in the node. The ionic currents were described as being generated through membrane conductances (see Figure 6). The sodium conductances and slow potassium conductance in the node were based on single channel conductances and channel densities. Single channel conductances of 13pS and 8pS were used for the sodium channels and slow potassium channels, respectively [37]. The nodal densities for the sodium and slow potassium channels were set to 1000 channels/μm^2 ^[37] and 100 channels/μm^2 ^[38], respectively. The ion conductance of the fast potassium current was based on the work of [16], who found a fast potassium conductance of 15nS and a capacitive load of 1.4pF on the nodal membrane. The conductance of the fast potassium current was set from an estimate of the membrane area [16], which was based on the nodal capacitance in experimental data and the nodal capacitance per square micrometer [39].

The nodal resting potential was kept stable by a current leak to the internode, and the internodal resting potential was determined from this relationship. The internodal resting potential was kept stable by a small internodal sodium leak conductance. The nodal persistent sodium conductance was set by the fraction of nodal sodium channels that would be persistent. Therefore, the total number of nodal sodium channels was kept constant for all simulations.

### Membrane kinetics

The non-linear membrane dynamics were based on human data [16]. The ionic current was given as: transient sodium current i_Nat _= G_Nat_m^3^h(E-E_Na_), persistent sodium current i_Nap _= G_Nap_p^3^(E-E_Na_), fast potassium current i_Kf _= G_Kf_n^4^(E-E_K_), and slow potassium current i_Ks _= G_Ks_s(E-E_K_). The fractional activations (m, h, p, n and s) were given by the differential equation:

dx/dt = α_x_(1-x)-β_x_x, for x = m, h, p, n, s

where α_m_, α_p_, α_n_, α_s _= A(E-B)/(1-exp((B-E)/C)); β_m_, α_h_, β_p_, β_n_, β_s _= A(B-E)/(1-exp((E-B)/C)), β_h _= A/(1+exp((B-E)/C)) (see Table 3 for the constant: A, B, and C) and E is the membrane potential. The rate constants (α_x _and β_x_) where scaled by appropriate Q_10 _factors to a temperature of 37°C (see Table 3). All membrane kinetics were the same as those of [16] except the kinetics for the slow potassium current (see section entitled 'Validation', below). In order to allow the model to reproduce threshold electrotonus, it was necessary to modify the slow potassium channels. The kinetics were changed in order to slow the channel activation and to lower the fraction of open slow potassium channels at the resting potentials for the node and internode.

**Table 3:** Rate constants

	**Q_10_**	**A**	**B**	**C**
		ms^-1^	mV	mV
α_m_	2.2	1.86	-18.4	10.3
β_m_	2.2	0.086	-22.7	9.16
α_h_	2.9	0.0336	-111.0	11.0
β_h_	2.9	2.3	-28.8	13.4
α_p_	2.2	0.93	-38.4	10.3
β_p_	2.2	0.043	-42.7	9.16
α_n_	3.0	0.00798	-93.2	1.1
β_n_	3.0	0.0142	-76.0	10.5
α_s_	3.0	0.0122	-12.5	16.9
β_s_	3.0	0.000736	-80.1	12.6

### Validation

The model was validated with four sets of experimental data: threshold electrotonus [40], recovery cycle [41], latent addition [10] and accommodation curve [5] (slope and breakdown of accommodation). Threshold electrotonus is important as it provides insight into internodal conductances in human subjects *in vivo*, and it is promising for providing insight into disease mechanisms in neurological disorders [42]. In threshold electrotonus, sub-threshold currents are used to alter the nodal and internodal membrane potentials. The change in threshold to a test stimulus is measured during the sub-threshold current, and this pattern of threshold alternations is termed threshold electrotonus [43]. The recovery cycle is a series of threshold fluctuations following an action potential. It is obtained by stimulating with a supra-threshold conditioning pulse and estimating the threshold with a subsequent test stimulus at various inter-stimulus intervals [43]. The threshold is usually tracked up to 200 ms after the conditioning pulse, during which time it goes through the absolute refractory period, relative refractory period, supernormal period and subnormal period. During the refractory and subnormal periods the threshold is increased, whereas it is decreased during the supernormal period [42,44]. Latent addition is obtained in the same manner as the recovery cycle [9,10]. The difference between the recovery cycle and latent addition is that the conditioning pulse is sub-threshold in latent addition but super-threshold in the recovery cycle. The strength-duration time constant τ was determined from the latent addition curve by fitting the function S2 = 100 - 90e^-s/τ ^to the simulated data, where S2 is the threshold of the test stimulus and s is the delay between the sub-threshold conditioning stimulus and the test stimulus. Eleven delays, equally spaced between 0.0 ms and 1.0 ms, were used in this fit (see [10] for a more detailed description of the estimation of the strength-duration time constant using latent addition). The accommodation curve is a plot of the threshold current as a function of the time-constant of current rise for exponentially rising stimuli [5]. Exponentially rising stimuli have the form I_S_(1-exp(-t/τ)), where τ is the time-constant of current rise.

Five parameters were adjusted in order to fit the model to these experimental data: the nodal resting potential, the internodal leak resistance, the internodal slow potassium conductance, the nodal persistent sodium conductance and the kinetics of the slow potassium channel. Throughout the paper, modeling data are presented as superimposed on the corresponding experimental ranges, a method taken from [24].

### Implementation

The model was implemented in C and integrated by Euler's method with a time step of 2 μs. The model was interfaced with Matlab 6.0 as a mex function, and m-functions were written to estimate measurements of axonal excitability. The excitability measurements were based on a binary search algorithm, which determined the excitation threshold with an accuracy of 0.1pA. An action potential was identified if the nerve fiber was depolarized to -30 mV with a rate of rise of more than 60 mV/ms. Stimulation was achieved by an intracellularly-injected current in the node.

## Competing interests

The author(s) declare that they have no competing interests.

## Authors' contributions

KH contributed extensively in all phases of the present study. OKA and LAN contributed to the planning of the study and to the discussion of the results.
